# Network meta‐analysis of multicomponent interventions

**DOI:** 10.1002/bimj.201800167

**Published:** 2019-04-25

**Authors:** Gerta Rücker, Maria Petropoulou, Guido Schwarzer

**Affiliations:** ^1^ Institute of Medical Biometry and Statistics Faculty of Medicine and Medical Center – University of Freiburg Freiburg Germany; ^2^ Department of Primary Education School of Education, University of Ioannina Ioannina Greece

**Keywords:** combination therapies, complex interventions, disconnected networks, multiple interventions, network meta‐analysis

## Abstract

In network meta‐analysis (NMA), treatments can be complex interventions, for example, some treatments may be combinations of others or of common components. In standard NMA, all existing (single or combined) treatments are different nodes in the network. However, sometimes an alternative model is of interest that utilizes the information that some treatments are combinations of common components, called component network meta‐analysis (CNMA) model. The additive CNMA model assumes that the effect of a treatment combined of two components A and B is the sum of the effects of A and B, which is easily extended to treatments composed of more than two components. This implies that in comparisons equal components cancel out. Interaction CNMA models also allow interactions between the components. Bayesian analyses have been suggested. We report an implementation of CNMA models in the frequentist R package **netmeta**. All parameters are estimated using weighted least squares regression. We illustrate the application of CNMA models using an NMA of treatments for depression in primary care. Moreover, we show that these models can even be applied to disconnected networks, if the composite treatments in the subnetworks contain common components.

## 1 INTRODUCTION

Meta‐analysis has evolved to a core method for summarizing evidence from multiple studies in medicine and healthcare. Network meta‐analysis (NMA) is an extension of pairwise meta‐analysis to compare three or more treatments for a given medical condition, based on combining information from multiple existing comparisons among subsets of the treatments (Bucher, Guyatt, Griffith, & Walter, 1997; Higgins & Whitehead, 1996; Lu & Ades, 2006, 2009; Lu, Welton, Higgins, White, & Ades, 2011; Lumley, 2002; Salanti, 2012).

Many healthcare treatments are complex interventions, for example, some treatments may be combinations of others or consist of common components. There have been attempts to define complexity for health interventions and many definitions have been suggested. Complex interventions are usually defined as consisting of several possibly interacting components, but definitions also described complex interventions that might require a large amount of organizational level, a large number and variability of outcomes, or a high degree of flexibility and tailoring of the interventions (Craig et al., 2008; Hawe, Shiell, & Riley, 2004; Kühne, Ehmcke, Härter, & Kriston, 2015; Petticrew, 2011; Petticrew et al., 2013). In this paper, we will concentrate on only one aspect, that of multicomponent interventions.

Welton, Caldwell, Adamopoulos, and Vedhara (2009) were the first authors who developed models for NMA of multicomponent interventions by considering their components. They classified psychological interventions for adults with coronary heart disease into five components of intervention (usual care only, educational, behavioral, cognitive, and support) and considered further 14 treatments that were combinations of two, three, or four of these basic components (Welton et al., 2009, table 2 there).

A standard approach to include multicomponent treatments in an NMA considers each unique combination of basic components as a distinct node in the network. An alternative approach aims at splitting each intervention into its components. Melendez‐Torres, Bonell, and Thomas (2015) introduced a different distinction between “building clinically meaningful units” and “components and dismantling.” A “clinically meaningful units” (or lumping, Caldwell & Welton, 2016) approach means combining a number of similar treatment modalities (e.g., all drugs within a class of substances, or a set of psychotherapies thought similar) into one treatment node. By contrast, a “components and dismantling” approach would seek to disentangle the common components of different treatments to identify their contribution to the effect of the combined intervention.

In this paper, we focus on the latter approach. Another example of data of this type was given by Mills, Druyts, Ghement, and Puhan (2011); Mills, Thorlund, and Ioannidis (2012) who presented an example of a network of 10 treatments for treatment of chronic obstructive pulmonary disease (COPD) (Mills et al., 2012, fig. 1 there). These 10 treatments consist of five components: inhaled corticosteroid (ICS), long‐acting betaagonist (LABA), long‐acting muscarinic agent (LAMA), phosphodiesterase‐4 inhibitor (PDE4‐i), and placebo, where placebo may be seen as a potentially inactive reference treatment. More recently, multicomponent analyses were presented by Caldwell and Welton (2016), Freeman et al. (2018), and Pompoli et al. (2018).

Bayesian approaches to analyze data of this type have been suggested (Welton et al., 2009) and applied (Freeman et al., 2018; Mills et al., 2011, 2012; Madan et al., 2014; Pompoli et al., 2018), also in a simulation study (Thorlund & Mills, 2012). A review of methods for meta‐analysis of complex health interventions is found in Tanner‐Smith and Grant (2018). A more general overview is given by Higgins et al. (2019).

The objective of this paper is to introduce a frequentist analysis approach to component network meta‐analysis (CNMA), which we implemented in the open source R package **netmeta** (R Core Team, 2018; Rücker, Krahn, König, Efthimiou, & Schwarzer, 2019). The paper is structured as follows. In Section 2, we introduce our data example, a real data set from a published NMA. In Section 3, after introducing the standard frequentist model for NMA in Subsection 3.1, we present an additive CNMA model (Subsection 3.2), show how it can be compared to the standard NMA model (Subsection 3.3), introduce interaction CNMA models (Subsection 3.4), and finally explain how CNMA models can be applied to connect disconnected networks (Subsection 3.5). The results for the example are shown in Section 4, and the paper ends with a discussion, Section 5.

## 2 DATA

In this paper, we will use a data set from an NMA of 22 treatments of depression in primary care (Linde, Rücker, Schneider, & Kriston, 2016), based on 100 trials in total with 21,298 patients in 217 treatment arms (79 two‐arm trials, 13 three‐arm trials, and one four‐arm trial). The primary outcome was response after treatment (yes/no), defined as a reduction from baseline by at least 50% on a depression scale. The data set using the odds ratio (OR) as effect measure is publicly accessible from the R package **netmeta** (Rücker et al., 2019, data set “Linde2016”).

The interventions comprised both medical and psychological treatments, also in combination, including placebo and usual care (UC) (Linde et al., 2016, fig. 1 there). Pharmacological interventions were tricyclic antidepressants (TCA), selective serotonin reuptake inhibitors (SSRI), serotonin‐noradrenaline reuptake inhibitors (SNRI), noradrenaline reuptake inhibitors (NRI), low‐dose serotonin (5‐HT2) antagonists and reuptake inhibitors (low‐dose SARI), noradrenergic and specific serotonergic agents (NaSSa), reversible inhibitors of monoaminoxidase A (rMAO‐A), hypericum extracts, and an individualized drug. Psychological interventions were cognitive behavioral therapy (CBT; 4 forms: face‐to‐face CBT, remote therapist‐led CBT, guided self‐help CBT, and no or minimal contact CBT), face‐to‐face problem‐solving therapy (PST), face‐to‐face interpersonal psychotherapy, face‐to‐face psychodynamic therapy, and “other face‐to‐face therapy.” Combination therapies were face‐to‐face CBT + SSRI, face‐to‐face PST + SSRI, and face‐to‐face interpersonal psychotherapy + SSRI.

## 3 METHODS

We propose the following procedure. First, a standard NMA is conducted where each possible combination of components is considered as a separate intervention and all existing single and combination treatments are different nodes in the network. Such a standard NMA is known as a full interaction model (Welton et al., 2009, Model 4). Second, based on combining the additive model with the network structure we obtain a model that describes how the observed treatment contrasts are combined from the components (Welton et al., 2009, Model 2). We show how the parameters of the additive CNMA model can be estimated. As a result, we obtain estimates for (a) the net effects of the components, compared to a reference treatment such as placebo; (b) the treatment effects, which are expressed as additive combinations of the components, again compared to the reference; and (c) estimates for all possible comparisons in the network, based on the network structure.

### 3.1 The standard NMA model

We follow the frequentist approach introduced by Rücker (2012). Let *m* be the number of pairwise treatment comparisons. In the special case of only two‐arm studies, this corresponds to the number of studies. More generally, each multi‐arm study with *p* arms contributes p(p−1)/2 comparisons and we have to adjust for multiarm studies (Rücker & Schwarzer, 2014). Accordingly, *m* is typically greater than the number of studies. Suppose we have *n* treatments, and let the n×1 vector θ represent the *n* treatment‐based (true) responses. We have data from *m* pairwise comparisons, denoted by $d=(d_{1},d_{2},\cdots ,d_{m})$ with associated standard errors $\text{SE}(d_{j}),\;j=1,\cdots ,m$. The $d_{j}$ may have been measured as mean differences, log risk ratios, log odds ratios, or other common effect measures. As usual in meta‐analysis, we assume the standard errors known and fixed. The model is

(1) d=Xθ+ε,ε∼N(0,Σ),

where **X** is the design matrix describing the structure of the network and Σ is a variance–covariance matrix. We may write this model briefly δ=Xθ where δ denotes the vector of true parameters for the contrasts. From now on we will use this notation.

Let **W** (the “weight matrix”) be a diagonal matrix of dimension m×m whose diagonal elements are weights $\left(w_{1},\cdots ,w_{m}\right)$. For two‐arm studies, the weights are the inverses of the observed variances, for multiarm studies they are assumed to be adjusted as described in Rücker and Schwarzer (2014). We can estimate the true parameters $\delta^{nma}$ as

$${\hat{\delta }}^{nma}=X{\left(X^{T}WX\right)}^{+}X^{T}Wd,$$

where ${\left(X^{T}WX\right)}^{+}$ is the Moore–Penrose generalized inverse (also called pseudoinverse (Albert, 1972; Rao & Mitra, 1971)) of the matrix $X^{T}WX$. The matrix $X^{T}WX$ is also called the Laplacian matrix (Rücker, 2012). The estimated variance–covariance matrix of ${\hat{\delta }}^{nma}$ is $X{\left(X^{T}WX\right)}^{+}X^{T}$.

(2) $$H=X{\left(X^{T}WX\right)}^{+}X^{T}W$$

is known in regression as the hat matrix.

### 3.2 The additive CNMA model

We begin by explaining the general idea of what we term CNMA, according to others (Freeman et al., 2018; Pompoli et al., 2018). To this aim, we consider a hypothetical example with three active treatment components *A*, *B*, and *C* (see Table 1). We consider five treatments: (a) *A* alone, (b) *A* combined with *B* (written A+B), (c) *A* combined with *B* and *C* (A+B+C), (d) *B* combined with *C* (B+C), and (e) placebo. Usually, we may conduct an NMA where all existing (single or combined) treatments are different nodes in the network. In the example, we have five nodes, corresponding to treatments (a) to (e). Perhaps, however, we are more interested in an alternative model that utilizes the information that treatments (a) to (d) are combinations of the elementary active components *A*, *B*, and *C*. The assumption is that the effects of combined treatments (here A+B,A+B+C,B+C) are additive sums of their components. This implies that in comparisons equal components cancel out. For the example, additivity means that

A+B versus *A* estimates *B*;
A+B+C versus *A* estimates B+C;
A+B+C versus A+B estimates *C*; and
A+B+C versus B+C estimates *A*.

Again, we consider a set of *n* treatments, which now may be combinations from a set of *c* clinically defined components, including a reference or “null” component, for example placebo. As before, the data consist of *m* pairwise comparisons of treatments from the set of treatments. Let each comparison j=1,⋯,m be represented by an observed (relative) treatment effect $d_{j}$ with standard error $\text{SE}(d_{j})$. For sake of clarity, we here ignore that there may be multiarm studies, however, adjustment for multiarm studies in the CNMA model works as described in Rücker and Schwarzer (2014) for the standard NMA model. We now define three matrices.

Matrix **B** has *m* rows (corresponding to the pairwise comparisons) and *n* columns (representing the treatments) and describes the structure of the network: it contains for each comparison in the network the entries 1 and −1 in the columns corresponding to the treatments compared, and zero entries otherwise. **B** corresponds to the edge–vertex incidence matrix as defined in Rücker (2012).

Matrix **C** is a n×c matrix describing how the *n* treatments are composed by the *c* active components, where an entry 1 indicates that the component in the column contributes to the treatment in the row, whereas zero entries indicate no contribution.

The design matrix of the additive model is

(3) $$X_{a}=BC.$$

For the hypothetical data in Table 1, ordering the studies (rows) by their number and the treatments (columns) by A,A+B,A+B+C,B+C, placebo, the network structure is described by

B=1−1−0−0−01−0−1−0−00−1−0−1−00−1−1−0−00−0−1−1−01−0−0−0−1.

The combination structure is described by

$$C=\left(\begin{array}{ccc} 1 & 0 & 0\\ 1 & 1 & 0\\ 1 & 1 & 1\\ 0 & 1 & 1\\ 0 & 0 & 0 \end{array}\right),$$

where the treatments (now represented by the rows) are ordered as above and the columns correspond to the components *A*, *B*, and *C*. Note that the reference treatment, here placebo, is not counted as a component, and therefore placebo as a treatment (last row) consists of no active component and all entries in this row are zero. The product provides the design matrix, where the rows represent the studies and the columns the components *A*, *B*, and *C*:

Xa=BC=−0−1−0−0−1−1−1−0−1−0−0−1−1−0−0−1−0−0.

**Table 1:** Hypothetical data

Study	Arm 1	Arm 2	Treatment effect	Standard error
Study 1	*A*	A+B	*d*_1_	SE(*d*_1_)
Study 2	*A*	A+B+C	*d*_2_	SE(*d*_2_)
Study 3	A+B	B+C	*d*_3_	SE(*d*_3_)
Study 4	A+B	A+B+C	*d*_4_	SE(*d*_4_)
Study 5	B+C	A+B+C	*d*_5_	SE(*d*_5_)
Study 6	*A*	Placebo	*d*_6_	SE(*d*_6_)

For ease of presentation, we here introduce the common (or fixed) effect additive CNMA model^1^ which is

(4) $$\delta_{a}=X_{a}\beta =BC\beta =B\theta_{a},$$

where $\delta_{a}\in R^{m}$ is the vector of true relative effects (differences) from the studies, $X_{a}=BC$ the design matrix, $\beta \in R^{c}$ a parameter vector of length *c*, representing the active components, and $\theta_{a}=C\beta \in R^{n}$ a vector of length *n*, representing the treatments. We want to obtain a weighted least squares estimate of β, using the inverse variance weights from the observed effects that we write as a diagonal matrix **W** of dimension m×m:

$$W={\left(\text{Cov}\;\left(d\right)\right)}^{-1}=\left(\begin{array}{ccccc} \sigma_{1}^{-1} & 0 & \cdots & \cdots & 0\\ 0 & \sigma_{2}^{-1} & 0 & \cdots & 0\\ : & : & : & : & :\\ 0 & \cdots & \cdots & \cdots & \sigma_{m}^{-1} \end{array}\right).$$

**Estimation of** β Using again the theory described in Rücker (2012), we obtain the estimate

(5) $$\hat{\beta }={\left(X_{a}^{⊤}WX_{a}\right)}^{+}X_{a}^{⊤}Wd$$

for the component effects β with estimated covariance matrix

$$\hat{\text{Cov}\;}\left(\hat{\beta }\right)={\left(X_{a}^{⊤}WX_{a}\right)}^{+}.$$

**Estimation of** $\theta_{a}$ The treatment effects $\theta_{a}$ are estimated by

$${\hat{\theta }}_{a}=C\hat{\beta }$$

with covariance matrix

$$\hat{\text{Cov}\;}\left({\hat{\theta }}_{a}\right)=C{\left(X_{a}^{⊤}WX_{a}\right)}^{+}C^{⊤}.$$

**Estimation of** $\delta_{a}$ The comparisons (contrasts) $\delta_{a}$ are estimated by

(6) $${\hat{\delta }}_{a}=X_{a}\hat{\beta }$$

with covariance matrix

$$\hat{\text{Cov}\;}\left({\hat{\delta }}_{a}\right)=X_{a}{\left(X_{a}^{⊤}WX_{a}\right)}^{+}X_{a}^{⊤}.$$

### 3.3 Heterogeneity, additivity test, and random effects CNMA

As the crucial additivity assumption may not hold, it is important to test whether this assumption is compatible with the data. To this aim, we propose a test of additivity which is based on the comparison of treatment estimates from the standard NMA model (1) and the additive CNMA model (4).

The hat matrix (2) of the standard NMA model, **H**, is a projection matrix that maps each vector in $R^{m}$ onto its image in the (n−1)‐dimensional subspace $S\subseteq R^{m}$ of consistent vectors (Rücker & Schwarzer, 2014). Particularly, **d** is mapped onto $Hd={\hat{\delta }}^{nma}\in S$. The space of vectors that are not only consistent in the sense of the standard NMA model, but also consistent with the CNMA model is a *c*‐dimensional subspace $C\subseteq S\subseteq R^{m}$ where *c* is the rank of the design matrix $X_{a}$. The projection matrix

$$H_{a}=X_{a}{\left(X_{a}^{⊤}WX_{a}\right)}^{+}X_{a}^{⊤}W$$

represents the corresponding hat matrix that maps the observed effects **d** onto their model‐based estimates $H_{a}d={\hat{\delta }}_{a}\in C\subseteq S$. Due to Hd∈S and $H_{a}d\in S$, we have also $Hd-H_{a}d\in S$. Because the two projections are commutative ($H_{a}H=H_{a}=H\;H_{a}$) we have

$${\hat{\delta }}_{a}=H_{a}d=H_{a}Hd=H_{a}{\hat{\delta }}^{nma}.$$

The heterogeneity statistic for the standard NMA model is

$$Q={\left(d-{\hat{\delta }}^{nma}\right)}^{⊤}\;W(d-{\hat{\delta }}^{nma}),$$

where ${\hat{\delta }}^{nma}$ denotes the vector of estimates based on the standard NMA model. Under standard conditions, *Q* follows a chi‐square distribution with $n_{a}-k-\left(n-1\right)$ degrees of freedom, where $n_{a}$ is the total number of treatment arms, *k* is the number of studies, and *n* is the number of treatments. If there are only two‐arm trials, we have $n_{a}=2k$ and thus k−(n−1) degrees of freedom.

The heterogeneity statistic for the CNMA model is

$$Q_{a}={\left(d-{\hat{\delta }}_{a}\right)}^{⊤}\;W(d-{\hat{\delta }}_{a}),$$

where ${\hat{\delta }}_{a}$ denotes the vector of estimates based on the additive treatment as given above. For $Q_{a}$, we have $df_{a}=n_{a}-k-r$ degrees of freedom where *r* is the rank of the design matrix $X_{a}$. If there are only two‐arm trials, $Q_{a}$ has k−r degrees of freedom.

We now provide a statistical test for the additivity assumption based on the Pythagorean theorem. Using the statistic

$$Q_{a}-Q={\left({\hat{\delta }}_{a}-{\hat{\delta }}^{nma}\right)}^{⊤}\;W\left({\hat{\delta }}_{a}-{\hat{\delta }}^{nma}\right)$$

with n−r−1 degrees of freedom we can test whether the (richer) standard NMA model (i.e., the model with more parameters) is superior to the sparser (i.e., more parsimonious) CNMA model (with fewer parameters), thus testing the assumption of additivity. The additive CNMA model will explain the data as well as the standard NMA model if no substantial unexplained heterogeneity exists.

A random effects CNMA model assuming a common between‐study variance τ^2^ can be implemented similar to Rücker and Schwarzer (2014) by using a multivariate methods of moments estimate of τ^2^ (Jackson, White, & Riley, 2012):

$${\hat{\tau }}^{2}=\max\left(\frac{Q_{a}-{\mathrm{df}}_{a}}{tr\left((I-H_{a})UW\right)},0\right)$$

with $Q_{a},df_{a},H_{a},W$ defined as above. **I** is the m×m identity matrix and tr denotes the trace of a matrix, that is, the sum of its diagonal elements. **U** is a block diagonal matrix derived from the m×m matrix $0.5\;BB^{⊤}$, obtained by selecting for each *p*‐arm study a p×p block, setting all other matrix elements to zero. The estimate ${\hat{\tau }}^{2}$ is added to the observed sampling variance of each single comparison in the network before adjusting the standard errors for multiarm studies and repeating the procedure described in Section 3.2 with the resulting enlarged standard errors.

### 3.4 The interaction CNMA model

The additive CNMA model assumes that there is no interaction between components and the effect of the combination of two or more treatment components is additive which may be clinically or biologically implausible. Furthermore, the test of additivity described in the previous subsection may suggest that the additive model does not fit the data well. Allowing interactions between pairs of clinically defined components, the additive model can be extended to the two‐way interaction CNMA model (Welton et al., 2009, Model 3). In the presence of an interaction the combination of components may act synergistically or antagonistically providing greater or smaller effects than the sum of their effects, respectively (Welton et al., 2009). For the frequentist approach, the interaction CNMA model is easily implemented by adding further columns to the combination matrix **C** of the additive CNMA model that represent interaction terms of interest.

In our hypothetical data example, an interaction term for the combination of treatments *A* and *B* would be represented by an additional (rightmost) column in the matrix **C** with 1 in each row belonging to a treatment that contains A+B, that is, treatments A+B and A+B+C in the second and third rows:

$$C_{int}^{A*B}=\left(\begin{array}{cccc} 1 & 0 & 0 & 0\\ 1 & 1 & 0 & 1\\ 1 & 1 & 1 & 1\\ 0 & 1 & 1 & 0\\ 0 & 0 & 0 & 0 \end{array}\right).$$

Other interaction terms could be added, however, as always, there is a trade‐off between model fit and sparseness. Furthermore, it makes no sense to add treatment combinations as interactions that do not occur in any study—even if they seem clinically plausible (corresponding columns would only contain zeros, indicating that these interactions cannot be estimated). This implicitly assumes that these interactions do not exist. Whether this assumption is justified for treatment combinations that are not represented in the given data cannot be tested. The richest sensible model is the standard NMA model where each unique combination has its own term in the model. Nested models can be compared using *Q* tests as described in the previous subsection. This allows disentangling the effects of all considered components, whether single components or interactions.

In practice, this is implemented in complete analogy to the additive CNMA model. Based on an extended combination matrix $C_{int}$ with one or more added column(s) for an interaction CNMA model, we obtain another design matrix $X_{int}$ by (3), and for estimation we use equations (4) to (6) accordingly. Testing for heterogeneity and comparing the standard model (that is, the full interaction model) to the chosen sparser interaction CNMA model works as described in Subsection 3.3, replacing $Q_{a}$ for the additive model with $Q_{int}$ for the interaction model. The chosen interaction CNMA model can also be compared to the even sparser additive model by comparing $Q_{int}$ and $Q_{a}$. We provide an example in the Results section.

### 3.5 CNMA models for disconnected networks

It may happen that a network is disconnected, which means that the set of treatments is partitioned in two or more subsets such that there is no study that compares a treatment in one subset to any treatment in another subset. Some approaches to disconnected networks have been suggested (Béliveau, Goring, Platt, & Gustafson, 2017; Goring et al., 2016), some based on arm‐based NMA models (Hawkins, Scott, & Woods, 2016; Hong, Chu, Zhang, & Carlin, 2016), others based on methods for population‐ (or matching‐)adjusted indirect comparisons (Phillippo et al., 2018; Signorovitch et al., 2012; Veroniki, Straus, Soobiah, Elliott, & Tricco, 2016).

We note that using CNMA models allows “reconnecting” a disconnected network if all subnets are connected to each other by treatments that have (potentially different) common components.

**Figure 1:**
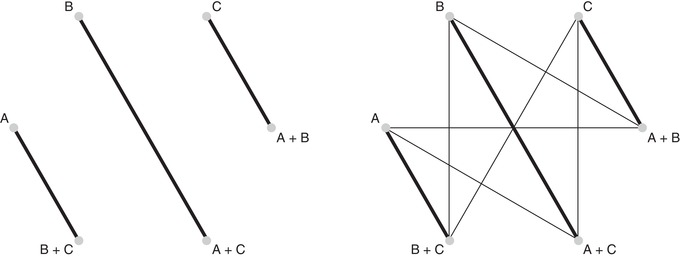
Left panel: A disconnected network of three two‐arm studies with six treatments. Right panel: The CNMA model adds new joins between the treatments having common components, thus reconnecting the network

A simple hypothetical example is a network of three two‐arm studies, one study comparing treatment *A* with B+C, another comparing *B* with A+C, and a third study comparing A+B with *C*. There are six treatments, and each study forms a subnetwork not connected to the others, all illustrated in Figure 1, left panel. However, all studies have common treatment components *A*, *B*, *C*, and their contributions can be estimated using the CNMA model, symbolized by the right part of Figure 1.

Ordering the treatments A,B,C,B+C,A+C,A+B, the network structure is described by

B=10−0−1−0001−0−0−1000−1−0−01,

the combination structure is

$$C=\left(\begin{array}{ccc} 1 & 0 & 0\\ 0 & 1 & 0\\ 0 & 0 & 1\\ 0 & 1 & 1\\ 1 & 0 & 1\\ 1 & 1 & 0 \end{array}\right),$$

and the design matrix becomes

Xa=BC=−1−1−1−1−1−1−1−1−1

with full rank 3. This means that all components A,B,C can be uniquely estimated. In this example, we do not specify an inactive treatment.

Even if we have only studies 1 and 3, some contrasts are still estimable. The four treatments are A,C,B+C,A+B. Matrices **B** and **C** reduce to

B=1−0−1−00−1−0−1,C=100001011110,

and the design matrix becomes

Xa=BC=1−1−11−1−1

with rank 2. If we are interested, for example, in the contrasts to *C*, we specify *C* as reference, thus omitting the last columns in **C** and $X_{a}$. This makes $X_{a}$ invertible, and by (5) and (6) we obtain unique estimates $\hat{\beta }$ for the contrasts *A* versus *C* and *B* versus *C*.

## 4 RESULTS

**Figure 2:**
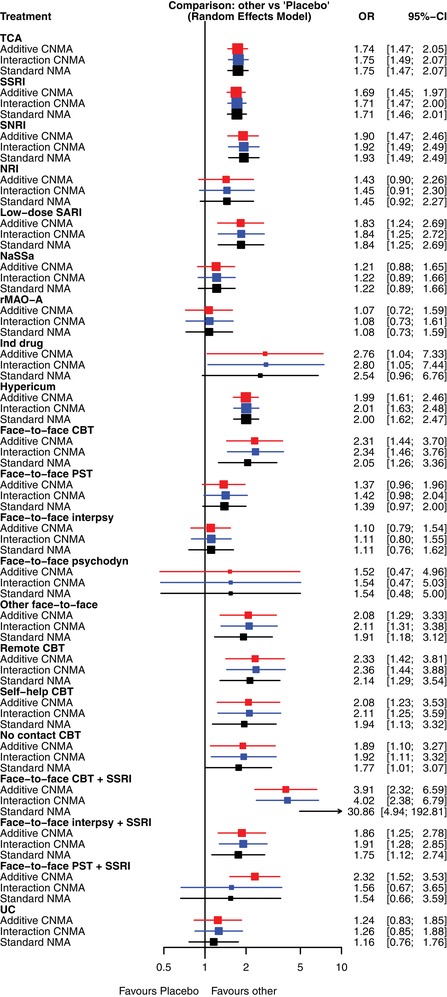
Comparing results of the additive model (red), an interaction model (blue), and the standard NMA model (black) for the depression data by a forest plot

We applied the standard random effects NMA model and two random effects CNMA models with and without an interaction term for face‐to‐face PST + SSRI to the depression data described in Section 2. For the primary outcome “response after treatment”, k=93 studies were available. The number of treatments was n=22. There were m=124 pairwise comparisons from 40 study designs, including 13 three‐arm studies and one four‐arm study. Placebo was chosen as reference for the standard NMA model and assumed as inactive for the two CNMA models. The results are shown in Table 2. They can be visualized by a forest plot (Figure 2).

**Table 2:** Results for the depression data (Linde et al., 2016)

Treatment (compared to placebo)	Standard model	Additive model	Additive model with one interaction^a^
	OR (95% CI)	OR (95% CI)	OR (95% CI)
TCA	1.75 [1.47–2.07]	1.74 [1.47–2.05]	1.75 [1.49–2.07]
SSRI	1.71 [1.46–2.01]	1.69 [1.45–1.97]	1.71 [1.47–2.00]
SNRI	1.93 [1.49–2.49]	1.90 [1.47–2.46]	1.92 [1.49–2.49]
NRI	1.45 [0.92–2.27]	1.43 [0.90–2.26]	1.45 [0.91–2.30]
Low‐dose SARI	1.84 [1.25–2.69]	1.83 [1.24–2.69]	1.84 [1.25–2.72]
NaSSa	1.22 [0.89–1.66]	1.21 [0.88–1.65]	1.22 [0.89–1.66]
rMAO‐A	1.08 [0.73–1.59]	1.07 [0.72–1.59]	1.08 [0.73–1.61]
Individualized drug	2.54 [0.96–6.76]	2.76 [1.04–7.33]	2.80 [1.05–7.44]
Hypericum	2.00 [1.62–2.47]	1.99 [1.61–2.46]	2.01 [1.63–2.48]
Face‐to‐face CBT	2.05 [1.26–3.36]	2.31 [1.44–3.70]	2.34 [1.46–3.76]
Face‐to‐face PST	1.39 [0.97–2.00]	1.37 [0.96–1.96]	1.42 [0.98–2.04]
Face‐to‐face interpsy	1.11 [0.76–1.62]	1.10 [0.79–1.54]	1.11 [0.80–1.55]
Face‐to‐face psychodyn	1.54 [0.48–5.00]	1.52 [0.47–4.96]	1.54 [0.47–5.03]
Other face‐to‐face	1.91 [1.18–3.12]	2.08 [1.29–3.33]	2.11 [1.31–3.38]
Remote CBT	2.14 [1.29–3.54]	2.33 [1.42–3.81]	2.36 [1.44–3.88]
Self‐help CBT	1.94 [1.13–3.32]	2.08 [1.23–3.53]	2.11 [1.25–3.59]
No contact CBT	1.77 [1.01–3.07]	1.89 [1.10–3.27]	1.92 [1.11–3.32]
Face‐to‐face CBT + SSRI	30.86 [4.94–192.81]	3.91 [2.32–6.59]	4.02 [2.38–6.79]
Face‐to‐face interpsy + SSRI	1.75 [1.12–2.74]	1.86 [1.25–2.78]	1.91 [1.28–2.85]
Face‐to‐face PST + SSRI	1.54 [0.66–3.59]	2.32 [1.52–3.53]	1.56 [0.67–3.65]
Usual care	1.16 [0.76–1.76]	1.24 [0.83–1.85]	1.26 [0.85–1.88]

a Interaction term for face‐to‐face PST + SSRI.

### 4.1 Results of the standard NMA model

For the standard model, heterogeneity and inconsistency were low (Q=102.45 with *df* = 87, p=.1234, between‐study variance $\tau^{2}=0.0174;I^{2}=15.1\%$). Decomposing *Q* into heterogeneity (within designs) and inconsistency (between designs) (Krahn, Binder, & König, 2013) provided $Q_{het}=58.07$ (*df* = 54, p=.3279) and $Q_{inc}=44.38$ (*df* = 33, p=.0892). The combination face‐to‐face CBT + SSRI was only assessed in one small two‐arm study (*n* = 34), compared to SSRI alone, where it showed an implausible large direct effect with a very long uncertainty interval (OR = 18 [2.95–109.66]) (Linde et al., 2015, 2016). This led to an even larger effect when comparing face‐to‐face CBT + SSRI to placebo (see Table 2).

### 4.2 Results of the additive CNMA model

The additive model was based on c=18 active components, corresponding to all treatments with the exceptions placebo and the three combinations of components face‐to‐face CBT, face‐to‐face interpsy, face‐to‐face PST with component SSRI. Note that we see the estimates for the components, relative to placebo, in the second column of Table 2. The three combinations were modeled as additive on the logit scale (and thus multiplicative on the OR scale). The estimated between‐study variance was $\tau^{2}=0.0208$ ($I^{2}=17.5\%$). The *Q* statistic for the additive model was $Q_{a}=109.12$ (*df* = 90, p=.0832) such that the difference $Q_{a}-Q=6.67$ (*df* = 3, p=.0831) suggested that the additive model explained the data quite well, so that the additivity assumption seems justified.

The biggest difference to the standard model was seen in comparison face‐to‐face CBT + SSRI versus placebo, where the additive model estimated an OR of 3.91 (which is the product of the ORs for face‐to‐face CBT vs. placebo (2.31) and SSRI vs. placebo (1.69)), whereas the standard model estimated an OR of 30.86. Thus the additive model had the (desired) effect of shrinking the implausibly large effect from the small study by borrowing strength from other studies that assessed one of the combined treatment's components.

Another difference to the standard model occurred with face‐to‐face PST + SSRI where the additive model provided a significant difference to placebo (OR of 1.37×1.69=2.32,p<.0001), potentially driven by the large effect of SSRI alone in the additive model, in contrast to the standard model that provided a nonsignificant 1.54 (p=.3136). This combination therapy was assessed in one three‐arm study with 151 participants, where it was compared to SSRI alone and to face‐to‐face PST alone. The proportion of treatment responses for the combination therapy lay between that by SSRI alone and that by face‐to‐face PST alone; none of the differences was statistically significant (*p*‐values .1242, .4095, and .5605).

### 4.3 Results of the interaction CNMA model

If we doubt the additivity assumption for face‐to‐face PST and SSRI, we may want to add an interaction between face‐to‐face PST and SSRI to the additive model. Allowing a clinically defined interaction between the face‐to‐face PST and the SSRI component, the interaction CNMA model can be implemented. Adding a column in matrix **C** indicating the interaction between face‐to‐face PST and SSRI, we have c=19. The results of this model are given in the rightmost column of Table 2. Most estimates tend to be very near to those of the standard NMA model, particularly, as expected, the comparison face‐to‐face PST + SSRI to placebo, for which an interaction is admitted. For the comparison face‐to‐face CBT + SSRI to placebo, the interaction model estimated an OR of 4.02 (standard model: 30.86), borrowing strength from other studies containing the components. Comparing the interaction CNMA model to the standard model, we obtained $Q_{int}-Q=5.55$ (*df* = 2, p=.0622), meaning that the interaction CNMA model was plausible and explained the data well. Comparing the interaction model with the additive model provided $Q_{a}-Q_{int}=1.12$ (*df* = 1, p=.29). The interpretation is that adding the interaction is not necessary here and the additive model is sufficient.

## 5 DISCUSSION

Although the use of NMA has considerably increased in the last couple of decades, CNMA for evaluating the effects of complex interventions and their components, though introduced already 10 years ago by Welton et al. (2009), seems not to be widely known and only occasionally applied. One possible explanation is the lack of easily accessible software. In this paper, we present a flexible frequentist implementation of the additive and the interaction CNMA models.

If there are treatments that are composed of common components, the additive model for CNMA allows (a) estimating effects of treatment components of combination therapies, (b) adding interaction terms by simply adding one column per interaction to the combination matrix **C**, and (c) comparing estimates and model fit between models, thus providing a statistical test for the additive or interaction model assumption using likelihood ratio statistics. An additive model has fewer parameters than the full interaction model (standard NMA model), which corresponds to a model that includes all observed interactions. CNMA models can be superior to the standard NMA model as they provide more powerful results while having fewer parameters to estimate (number of components instead of the number of observed combination of components). Furthermore, they allow borrowing strength from studies having common components for combinations that were evaluated in only a few studies or in only one small study at all. A simulation study has shown that if the additivity assumption approximately holds, the additive effects model was preferable to the conventional NMA (Thorlund & Mills, 2012). A case study with 51 observed combinations of 12 therapeutic components for panic disorder was published by Pompoli et al. (2018), giving more powerful results with the implementation of CNMA.

A possible objection to additive models is that they could mislead researchers to add a treatment to itself. In fact, one might ask whether, in rare cases, additive models are suitable to capture dose effects. If it seems justifiable to assume that, for example, a doubled dose of some drug *A* or a doubled duration of a treatment has about the double effect, this could be modeled by entering 2 in all rows of the *A* column of the combination matrix **C** that correspond to treatment combinations that include the doubled dose.

In our example, use of the additive CNMA resulted in a plausible shrinkage of the extreme face‐to‐face CBT + SSRI effect, which was evaluated only in one small study and constituted a separate node in the standard NMA model. However, we also observed a notable increase in the face‐to‐face PST + SSRI effect even resulting in a statistically significant effect that was not present in the standard NMA model. This motivated us to conduct a sensitivity analysis by considering this combination as an interaction term. The *Q* test revealed that the model with the single interaction was not significantly superior to the additive CNMA model.

In our application, we assume that placebo is an inactive treatment, implying that adding placebo to any active treatment does not change the effect of the respective treatment or, in other words, that comparing a treatment to placebo directly provides the treatment's net effect. Without this assumption, a nonnull treatment response for placebo would be estimated which we do not think meaningful in our depression example. A CNMA model assuming placebo as an active component would result in slightly different treatment estimates. We would like to emphasize the difference between choosing a treatment as the reference (which is merely a matter of parameterization) and assuming a treatment as inactive (which is an additional modeling assumption). We can use an active treatment as the reference, for example, to express treatment estimates relative to SNRI in the depression data set, however, it makes no sense to take an active treatment like SNRI as inactive.

Standard considerations for model selection apply if interaction CNMA models are used. The additive CNMA model without interactions has the smallest number of parameters to estimate while the standard NMA model has the most parameters, as it implicitly includes *all estimable* interactions which are prespecified by the network structure. Obviously, we could either start with the additive CNMA model and add interactions (corresponding to forward selection) or start with the standard NMA model and remove estimable interactions (backward selection); both approaches will lead to an interaction CNMA model between the two extremes. From a practical point, it is much easier in the CNMA setting to use forward than backward selection: we simply have to add a single column to matrix **C** instead of determining all estimable interactions and add all corresponding columns. In order to avoid data dredging, the interaction terms considered in the model selection should ideally be prespecified based on subject matter knowledge.

A special feature of CNMA models is its potential to connect a disconnected network, given the additivity assumption holds and the subnetworks have at least one common treatment component. This property seems to have been mostly overlooked (or at least not explicitly mentioned) in the literature, with the exception of Mawdsley, Bennetts, Dias, Boucher, and Welton (2016) who mention it in the context of meta‐analysis of dose–response curves (Mawdsley et al., 2016, p. 400). We gave a hypothetical example in Subsection 3.5. We note, however, that in a disconnected network the additivity assumption is not testable, as no standard NMA model exists which is necessary for comparison. We also point out that precision may not always increase when using a CNMA model, compared to a standard NMA. This holds particularly for disconnected networks. Nevertheless it may be worth the effort to connect a disconnected network, at the expense of losing some precision for comparisons from the same subnet.

We implemented the additive and interaction CNMA model in the functions netcomb (for connected networks) and discomb (for disconnected networks) of R package **netmeta** (Rücker et al., 2019). The application of **netmeta** is quite convenient: a command like netcomb(net1) is sufficient to conduct an additive CNMA for an existing R object, here called net1, with the results of the standard NMA. Accordingly, this frequentist implementation can be easily applied by researchers without extended training in statistical software. R code for the examples from this paper is provided in the supplementary material and also contained in the help files of netcomb and discomb. In contrast, the more flexible Bayesian CNMA implementation in WinBUGS is less accessible to the typical meta‐analyst. This is obvious by looking at the WinBUGS code in the supplementary material that we used to rerun all models for the depression data. In general, results using WinBUGS (see web supplement) were similar to those of our models (Table 2 and supplementary material). We observe the largest difference in the indirect estimate of face‐to‐face CBT + SSRI compared to placebo in the standard NMA model: log odds ratio 3.43 [1.60 – 5.26] (netmeta) and 3.61 [1.91 – 5.86] (WinBUGS). However, this estimate is based on one very small study comparing face‐to‐face CBT + SSRI with SSRI alone and the confidence intervals for the indirect comparison overlap to a very large extent.

In conclusion, we recommend to consider using a CNMA model, either a simple additive model or one with interactions, if multicomponent interventions occur in a meta‐analysis and either full additivity or additivity with some added interactions is assumed. These models can now be analyzed in a frequentist framework, and with the R package **netmeta** an open source software to this aim is readily available.

## CONFLICT OF INTEREST

The authors have declared no conflict of interest.

## Supporting information

Supporting Information

Supporting Information

## ENDNOTE

1 A random effects version is described in the next subsection.
