# Supplementary material for: Network meta‐analysis of multicomponent interventions
Source: Biom J. 2019 Apr 25;62(3):808–21. doi: 10.1002/bimj.201800167 (PMC7217213; doi:10.1002/bimj.201800167)
Supplement: Supplementary file 1 — Supporting Information [file BIMJ-62-808-s002.pdf]

Bayesian analysis

For the data set with 22 treatments of depression in primary care (Linde et al., 2016), we conducted Bayesian random-effects standard NMA, additive CNMA, and interaction CNMA using WinBUGS. In all Markov Chain Monte Carlo (MCMC) simulations, we assumed vague priors for treatment parameters and a half-normal prior distribution for the between-study standard deviation ($\tau \sim N(0,1)$, $\tau > 0$). The models were run for 100,000 iterations, after discarding the first 10,000 iterations.

The table provides the results of Bayesian analyses for the depression data (Linde et al., 2016) and reports the posterior median values of odds ratios with 95% credible intervals (CrIs) for the treatment estimates compared to placebo and the median value for heterogeneity.

In general, standard NMA, additive CNMA, and interaction CNMA yield similar results with the frequentist and Bayesian approach. For example, the ORs for the comparison of SSRI with placebo are 1.71 (standard NMA), 1.69 (additive CNMA), and 1.71 (interaction CNMA) under the frequentist approach compared to the results for the Bayesian approach: 1.72, 1.73, and 1.72, respectively. The largest difference between frequentist and Bayesian approach is observed for the comparison of face-to-face CBT + SSRI to placebo: 30.86 vs 36.94 (standard NMA), 3.91 vs 4.36 (additive CNMA), and 4.02 vs 4.28 (interaction CNMA).

In summary, treatment estimates and heterogeneity estimator from frequentist approach are similar to those of the Bayesian approach for the three models (standard NMA, additive CNMA, and interaction CNMA).

* Please note that standard WinBUGS codes do not offer calculating credible intervals for estimates of treatment combinations. Treatment estimates and their credible intervals for the three combination treatments (Face-to-face CBT + SSRI, Face-to-face interpersonal psychotherapy + SSRI, Face-to-face PST + SSRI) compared to placebo are based on the following equations.

Let us have the treatments A and B and let C be an inactive control treatment. Then the odds ratio for A+B compared to treatment C is

$$OR_{A+B\ versus\ C} = OR_{A\ versus\ C} * OR_{B\ versus\ C}$$

and the log odds ratio (LOR) is

$$LOR_{A+B\ versus\ C} = LOR_{A\ versus\ C} + LOR_{B\ versus\ C}$$

with lower confidence limit

$$l_{LOR_{A+B\ versus\ C}} = LOR_{A+B\ versus\ C} - 1.96 * SE(LOR_{A+B\ versus\ C})$$

and upper confidence limit

$$u_{LOR_{A+B\ versus\ C}} = LOR_{A+B\ versus\ C} + 1.96 * SE(LOR_{A+B\ versus\ C})$$

where $SE(LOR_{A+B\ versus\ C})$ is the standard error of the observed mean $LOR_{A+B\ versus\ C}$

$$SE(LOR_{A+B\ versus\ C}) = sqrt\big(SE(LOR_{A\ versus\ C})^2 + SE(LOR_{B\ versus\ C})^2 + 2 * \rho * SE(LOR_{A\ versus\ C}) * SE(LOR_{B\ versus\ C})\big)$$

with correlation coefficient ρ between $LOR_{A\ versus\ C}$ and $LOR_{B\ versus\ C}$ and standard errors $SE(LOR_{A\ versus\ C}), SE(LOR_{B\ versus\ C})$ of the observed means $LOR_{A+B\ versus\ C}$, $LOR_{B\ versus\ C}$, respectively.

The credible intervals on the odds ratio scale for treatment A+B compared to treatment C were back-transformed using $\exp\big(LOR_{A+B\ versus\ C} \pm 1.96 * SE(LOR_{A+B\ versus\ C})\big)$.

Table Bayesian Analysis for the depression data (Linde et al., 2016)

**treatment compared to placebo**	**Standard model**	**Additive model**	**Interaction model**
	**OR [95% CrI]**	**OR [95% CrI]**	**OR [95% CrI]**
TCA	1.76 [1.48, 2.10]	1.78 [1.49, 2.13]	1.77 [1.48, 2.13]
SSRI	1.72 [1.45, 2.05]	1.73 [1.46, 2.03]	1.72 [1.45, 2.04]
SNRI	1.94 [1.47, 2.53]	1.94 [1.47, 2.55]	1.93 [1.46, 2.55]
NRI	1.46 [0.89, 2.38]	1.46 [0.89, 2.38]	1.45 [0.88, 2.41]
Low-dose SARI	1.83 [1.25, 2.75]	1.85 [1.25, 2.77]	1.84 [1.23, 2.76]
NaSSA	1.21 [0.87, 1.67]	1.21 [0.88, 1.71]	1.21 [0.87, 1.66]
rMAO-A	1.08 [0.72, 1.62]	1.08 [0.72, 1.64]	1.08 [0.72, 1.64]
Individualized antidepressant	2.63 [0.94, 7.20]	2.98 [1.10, 8.09]	2.95 [1.09, 8.28]
Hypericum	2.03 [1.65, 2.55]	2.04 [1.65, 2.54]	2.05 [1.65, 2.55]
Face-to-face CBT	2.08 [1.29, 3.45]	2.52 [1.55, 4.13]	2.48 [1.52, 4.09]
Face-to-face PST	1.40 [0.95, 2.07]	1.41 [0.98, 2.06]	1.44 [0.99, 2.11]
Face-to-face interpersonal psychotherapy	1.13 [0.78, 1.66]	1.14 [0.78, 1.73]	1.11 [0.75, 1.70]
Face-to-face psychodynamic therapy	1.51 [0.48, 4.73]	1.59 [0.46, 5.05]	1.55 [0.45, 5.11]
Other face-to-face psychosocial therapies	1.95 [1.26, 3.19]	2.22 [1.36, 3.70]	2.19 [1.35, 3.71]
Remote therapist-lead CBT	2.17 [1.36, 3.64]	2.49 [1.51, 4.30]	2.47 [1.49, 4.25]
Guided self-help CBT	1.98 [1.14, 3.45]	2.23 [1.29, 3.89]	2.19 [1.27, 3.89]
No/minimal contact CBT	1.79 [1.05, 3.21]	2.02 [1.14, 3.63]	1.98 [1.13, 3.59]
Face-to-face CBT + SSRI	36.94 [6.72, 350.80]	4.36 [2.49, 7.62]*	4.28 [2.44, 7.55]*
Face-to-face interpersonal psychotherapy + SSRI	1.79 [1.11, 2.83]	1.98 [1.23, 3.22]*	1.92[1.17, 3.18]*
Face-to-face PST + SSRI	1.56 [0.67, 3.82]	2.45 [1.56, 3.84]*	2.49 [1.57, 3.95]*
Usual care	1.17 [0.78, 1.77]	1.31 [0.86, 2.04]	1.30 [0.85, 2.02]
Heterogeneity τ^2	0.0200	0.0218	0.0244

* Credible intervals for estimates of treatment combinations calculated as described in the text.
